# Case Report: Idiopathic intramural hematoma of the jejunum in a French Bulldog

**DOI:** 10.3389/fvets.2025.1577989

**Published:** 2025-05-20

**Authors:** Yeon-Jin Kim, Dong-Min Choi, Jong-Il Kang, Dong-Yeong Kim, Chan-Sik Nam, Kwang-Sup Lee, Hee-Myung Park

**Affiliations:** ^1^Department of Veterinary Internal Medicine, College of Veterinary Medicine, Konkuk University, Seoul, Republic of Korea; ^2^Choonghyun Animal Hospital, Seoul, Republic of Korea

**Keywords:** intramural hematoma, idiopathic, diarrhea, vomiting, case report, French bulldog, jejunum

## Abstract

This case report describes clinical and diagnostic features of an idiopathic jejunal intramural hematoma in a dog presenting with non-specific gastrointestinal symptoms. A 2-year-old neutered male French Bulldog was presented with vomiting, abdominal pain, and chronic soft stools persisting for 2 weeks, with no history of trauma or dietary indiscretion. Physical examination revealed abdominal distension and palpable discomfort. The complete blood count (CBC) showed leukocytosis with neutrophilia, accompanied by signs of dehydration. Serum chemistry revealed no significant abnormalities, although mild electrolyte imbalances were observed. The canine pancreatic lipase immunoreactivity (cPLI) test was within the reference range and the C-reactive protein (CRP) level was slightly elevated. Ultrasonography revealed a heterogeneous mass in the jejunal region, which was suspected to be a hematoma. An exploratory laparotomy was performed, and the affected jejunal segment was surgically resected. Histopathological examination confirmed the mass as an intramural hematoma. Additionally, coagulation profiles revealed no remarkable findings. The cause of the hematoma was determined to be idiopathic after excluding other possible causes. This case highlights the importance of considering intramural hematomas in the differential diagnosis for dogs presenting with non-specific gastrointestinal symptoms, even in the absence of trauma and coagulation abnormalities.

## Introduction

Chronic diarrhea is a common clinical symptom in dogs, resulting from either primary or secondary causes. Primary enteropathies include infectious, neoplastic, mechanical, toxic, and non-infectious inflammatory diseases. In contrast, secondary enteropathies are associated with systemic diseases affecting the pancreas, liver, kidneys, endocrine system, cardiovascular system, and central nervous system (1). According to a previous study published in 2017 (2), 90% of dogs presenting with chronic diarrhea were diagnosed with primary enteropathy, of which 79% were classified as chronic inflammatory enteropathy (CIE). Among the different forms of CIE, food-responsive enteropathy was the most prevalent, followed by antibiotic-responsive enteropathy and idiopathic inflammatory bowel disease. Neoplastic causes including intestinal lymphoma and intestinal adenocarcinoma were identified in only 4% of the cases.

Intestinal hematomas are well-documented in human medicine, with traumatic abdominal injury being the primary cause. Other reported etiologies include pancreatic diseases, anticoagulant therapy, intestinal biopsy, or injections (3). In contrast, occurrences of intramural hematomas in companion animals have been extremely rarely reported (4–7). To date, only five cases in dogs and one case in a cat have been reported across four studies. Among the reported canine cases, two were associated with chronic pancreatitis, one was related to a foreign body, and two were classified as undetermined. The single feline case involved a colonic hematoma of unknown origin (4–7).

Therefore, given the rarity of such cases, this case report aims to document the diagnostic process of an idiopathic jejunal intramural hematoma in a 2-year-old castrated male French Bulldog, emphasizing the importance of including intramural hematoma in the differential diagnosis for dogs presenting with non-specific gastrointestinal symptoms, even in the absence of trauma and coagulation abnormalities.

## Case description

A 2-year-old neutered male French Bulldog was presented with clinical signs of vomiting, abdominal pain, and chronic soft stools persisting for 2 weeks. The owner reported no dietary indiscretions, recent trauma, or administration of any medications. Physical examination revealed that the dog was alert but lethargic, with mildly dry mucous membranes and a capillary refill time of 2 s. Rectal temperature was 38.5°C. The heart rate was 120 beats per minute, and respiratory rate was 24 breaths per minute, both within normal limits. Mild abdominal distension and discomfort on palpation were also noted in the mid-abdomen. No external signs of trauma, such as bruising, lacerations, or abrasions, were observed. To facilitate the differential diagnosis of these gastrointestinal symptoms, hematological and biochemical profiles were performed to rule out systemic diseases.

The complete blood count showed mild leukocytosis (12.7 × 10^9^/L; reference range: 6–12), with neutrophilia (11.6 × 10^9^/L; reference range: 3–10), elevated hemoglobin levels (20.2 g/dL; reference range: 12–18), and an increased hematocrit (60.7%; reference range: 37–55). The red blood cell count was at the upper limit of the reference range (8.49 × 10^12^/L; reference range: 5.5–8.5), while the platelet count remained within normal limits (363 × 10^9^/L; reference range: 200–500). Serum chemistry revealed no significant abnormalities except for a slightly elevated C-reactive protein (CRP) level at 7 mg/L (reference range: 0–7). Mild electrolyte imbalances were noted, with hyponatremia (141 mmol/L; reference range: 145–151), hypokalemia (3.63 mmol/L; reference range: 3.9–5.1), and hypochloremia (102 mmol/L; reference range: 110–119). These findings suggested no significant systemic abnormalities but indicated mild dehydration and electrolyte imbalances, likely due to gastrointestinal losses. Additionally, the canine pancreatic lipase immunoreactivity (cPLI) test was performed due to the chronicity of gastrointestinal symptoms persisting for 2 weeks. The results were within the normal range (<50 ng/mL; reference range: 0–200) (Table 1).

**Table 1:** Results of a complete blood count (CBC), serum chemistry, and electrolytes (WBC = 12.7 × 10^9^/L [6–12], NEU = 11.6 × 10^9^/L [3–10]).

Parameters	Results	Reference range
Complete blood count (CBC)		
RBC (M/uL)	8.49	5.5–8.5
HCT (%)	**60.7**	37.0–55.0
HGB (g/dL)	**20.2**	12–18
PLT (K/μL)	363	200–500
Serum Chemistry		
GLU (mg/dL)	119	75–128
CREA (mg/dL)	0.66	0.4–1.4
BUN (mg/dL)	13.5	9.2–29.2
TP (g/dL)	6.5	5–7.2
ALB (g/dL)	3.7	2.6–4.0
GLOB (g/dL)	2.8	1.6–3.7
ALT (U/L)	70	17–78
AST (U/L)	35	17–44
ALP (U/L)	146	47–254
CRP (mg/L)	7	0–7
cPLI (ng/ml)	<50	0–200
Electrolytes		
Na^+^ (mmol/L)	**141**	145–151
K^+^ (mmol/L)	**3.63**	3.9–5.1
Cl^−^ (mmol/L)	**102**	110–119

RBC, red blood cells; HCT, hematocrit; HGB, hemoglobin; PTL, platelet; GLU, glucose; CREA, creatinine; BUN, blood urea nitrogen; TP, total protein; ALB, albumin; GLOB, globulin; ALT, alanine aminotransferase; AST, aspartate aminotransferase; ALP, alkaline phosphatase; CRP, c-reactive protein; cPLI, canine pancreatic lipase immunoreactivity; Na^+^, sodium Ion; K^+^, potassium Ion; Cl^−^, chloride Ion. Bold values indicate abnormal findings.

**Figure 1:**
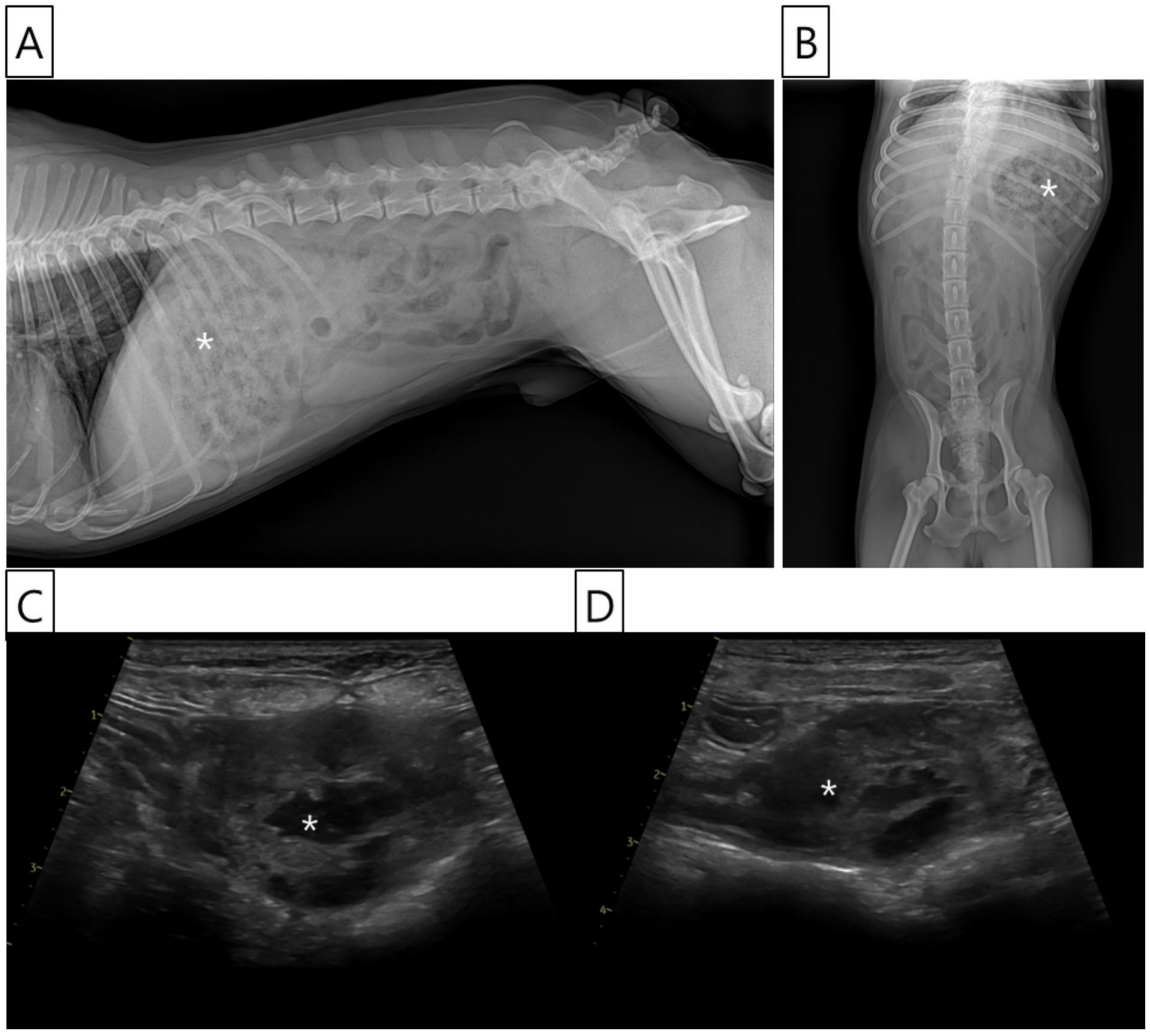
Radiographic and ultrasonographic images of the abdomen. **(A)** Right lateral abdominal radiograph showing food retention in the stomach (asterisk) and multiple gas-filled loops of the small intestine. However, no radiographic abnormalities suggestive of an intestinal lesion are identified. **(B)** Ventrodorsal abdominal radiograph also demonstrates food retention within the stomach (asterisk) and some intestinal gas patterns, but no specific abnormalities indicative of intestinal pathology. **(C, D)** Ultrasonographic images of the abdomen reveal a heterogeneous mass (asterisk) in the jejunal area, distal to the duodenum, measuring approximately 3.5 cm in length. The mass exhibited mixed echogenicity with internal fluid retention and showed an indistinct demarcation from the bowel wall layers. Loss of the normal wall structure was observed within the mass. However, no evidence of small bowel perforation or ascites was observed.

Radiographic examination indicated the presence of food retention within the stomach, with no significant abnormalities noted in the intestinal tract or other abdominal organs (Figures 1A,B). Ultrasound examination was performed using a Vivid E90 ultrasound system (GE Healthcare, Chicago, IL, USA) equipped with a 9–12 MHz linear transducer. Ultrasonography revealed a 3.5 cm mass in the distal duodenum, which was not well demarcated from the intestinal wall. The mass exhibited heterogeneous echogenicity and fluid accumulation observed within the lesion, consistent with features of an intramural lesion, as shown in the ultrasound image. No ultrasonographic signs of perforation or free abdominal fluid were observed (Figures 1C,D). Subsequently, computed tomography (CT) was recommended to assess tumor staging and potential metastasis; however, the owner declined further anesthesia or sedation solely for diagnostic purposes, and thus CT imaging was not performed.

Due to the size and obstructive potential of the jejunal mass identified on ultrasound, the dog owner consented to an exploratory laparotomy for surgical resection of the intramural jejunal mass. The patient was premedicated with cephradine (20 mg/kg IV; Hankook Korus Pharm, Chuncheon, Korea), tramadol (1.25 mg/kg IV; Jeil Pharm, Daegu, Korea), maropitant (1 mg/kg IV; Cerenia®, Zoetis, Parsippany, NJ, USA), and glycopyrrolate (0.01 mg/kg IV; Myungmoon Pharm, Seoul, Korea). Anesthesia induction was performed using propofol (1 mg/kg IV; Daewon Pharm, Seoul, Korea), and anesthesia was maintained with isoflurane (Forane®, Hana Pharm, Seoul, Korea) in 100% oxygen. The concentration of isoflurane was individualized to 1–3%, maintaining minimum alveolar concentration (MAC) of 1.3–1.5. During the surgical procedure, particularly during the more painful stages such as intestinal resection and anastomosis, more than 2% of isoflurane was administered.

**Figure 2:**
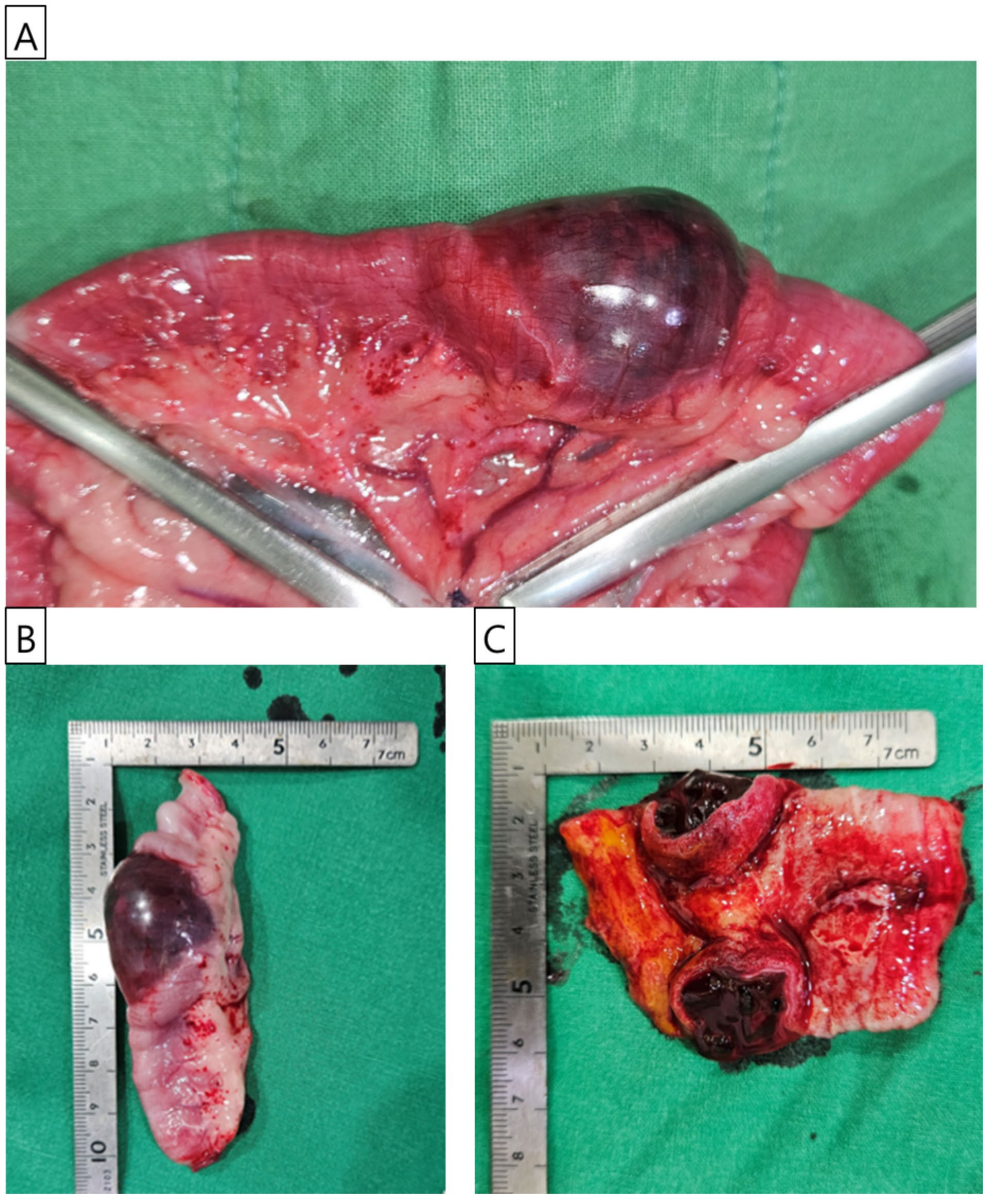
Gross features of the jejunal mass. **(A)** Intraoperative photograph showing the jejunal mass identified during surgery. The mass’s appearance was consistent with hematoma. The proximal and distal sections of the jejunum were secured with atraumatic intestinal clamp forceps before resection. **(B)** The excised jejunal mass, approximately 3.5 cm in size, is prepared for histopathological examination. **(C)** The bisected jejunal mass, examined for internal contents, reveals clotted blood within the lesion.

Surgical intervention was undertaken with the dog positioned in dorsal recumbency. A standard midline celiotomy was carried out, extending from approximately 4–5 cm caudal to the xiphoid process to 4–5 cm caudal to the umbilicus. Upon exploratory laparotomy, no gross lesions suggestive of trauma were identified in the abdominal organs or along the abdominal wall. Additionally, there were no observable abnormalities such as bowel wall edema, mesenteric thickening, or venous and lymphatic congestion within the mesentery. However, an intramural hematoma was identified in the jejunal segment. The affected jejunal loop was exteriorized and isolated from adjacent. The mesenteric vessels supplying the segment designated for resection were ligated using 2–0 monofilament absorbable suture (PDS II, Ethicon, Somerville, NJ, USA). A surgical margin of at least 4 cm was secured on both the proximal and distal sides of the lesion. Atraumatic intestinal clamps were placed approximately 6 cm proximal and distal to the lesion to temporarily occlude the intestinal lumen (Figure 2A). Stay sutures were placed on the antimesenteric borders of the proximal and distal intestinal loops near the intended transection sites using 3–0 polypropylene suture (Prolene, Ethicon, Somerville, NJ, USA) to facilitate alignment. The mass and adjacent tissues were subsequently resected. An end-to-end, appositional anastomosis was performed using 3–0 monofilament absorbable suture (PDS II, Ethicon, Somerville, NJ, USA). The anastomosis was initiated by placing three simple interrupted sutures in a triangular pattern to align the lumen, followed by a simple continuous pattern to complete the closure. A leaking test was conducted following anastomosis, and no leakage was observed. Omentum draping was performed over the anastomotic site following lavage with sterile saline to promote healing. Routine three-layer closure of the abdominal wall was performed. The linea alba was closed with simple interrupted sutures using 2–0 monofilament absorbable suture (PDS II, Ethicon, Somerville, NJ, USA). The subcutaneous tissues were approximated using 3–0 monofilament absorbable suture (Monocryl, Ethicon, Somerville, NJ, USA) in a simple interrupted pattern. Finally, the skin was closed using 3–0 non-absorbable monofilament suture (Prolene, Ethicon, Somerville, NJ, USA) in a simple interrupted pattern. After the anastomosis, the dog recovered well from anesthesia without any postoperative complications. The excised jejunal mass, measuring 3.5 cm in length, was prepared for histopathological evaluation (Figure 2B). Blood clots were observed in the dissected mass (Figure 2C).

**Figure 3:**
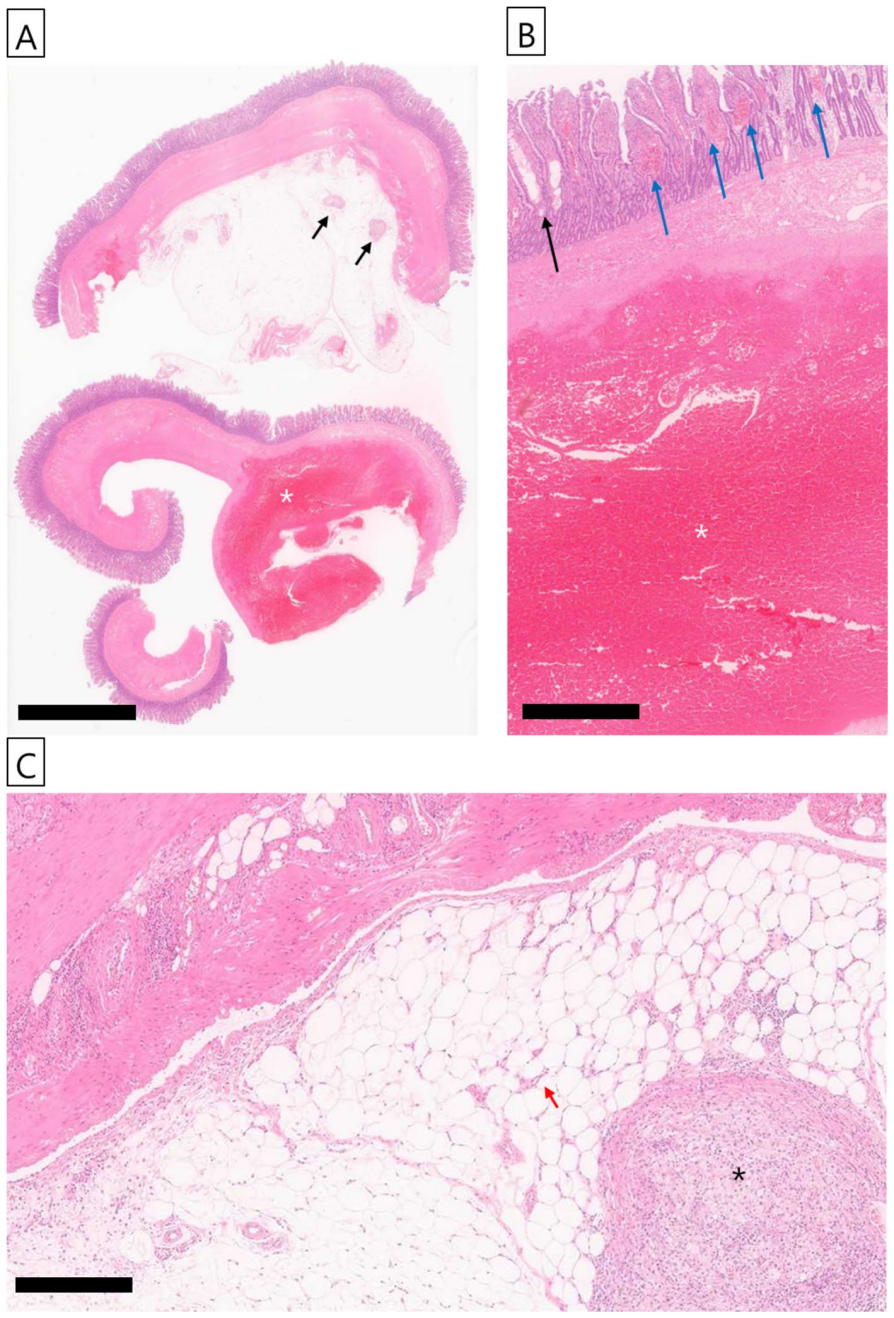
Histopathological examination of the mass. **(A)** A substantial portion of the muscular layer was extensively composed of retained blood (white asterisk), and granulomatous inflammation (black arrow) was observed in the mesentery; H&E stain; scale bar, 500 μm. **(B)** In the mucosal layer, lymphatic vessel dilation (black arrow) and congestion (blue arrow) were noted, with retained blood also evident in the muscular layer (white asterisk); H&E stain; scale bar, 100 μm. **(C)** In the mesentery, granulomatous inflammation (black asterisk) was observed, along with increased connective tissue and the formation of some newly developed blood vessels (red arrow). No other notable findings, such as infectious pathogens or neoplastic changes, were observed; H&E stain; scale bar, 100 μm.

The excised jejunal mass was submitted to Kopath Co., Ltd. (Seoul, Republic of Korea) for histopathological evaluation. Histological examination revealed extensive blood retention within a substantial portion of the muscular layer (Figure 3A), while the mucosal layer exhibited lymphatic dilation and blood congestion (Figure 3B). In the mesentery, granulomatous inflammation, increased connective tissue, and the formation of newly developed blood vessels were observed (Figure 3C). However, no other remarkable findings, such as infectious pathogens or neoplastic changes, were identified.

Potential causes of hematoma in the intestine, such as anticoagulant use, traumatic injury, foreign body, intussusception, and coagulopathy were considered. However, in this case, there was no history of anticoagulant use or traumatic injury, and given the dog’s young age, neoplastic processes were also unlikely. Additionally, no lesions suggestive of foreign body or intussusception were identified during exploratory laparotomy or histopathological examination, allowing these causes to be ruled out. Consequently, coagulation testing was warranted to evaluate platelet function and potential coagulation factor deficiencies, including von Willebrand disease (vWD). Since the platelet count was normal on pre-surgical testing, thrombocytopenia was ruled out as a cause. Postoperative evaluations of prothrombin (PT) and activated partial thromboplastin time (APTT) were within normal ranges, and the vWD factor test results were also normal. Therefore, this case was diagnosed as a hematoma caused by idiopathic etiology. For 6 months follow-up period, there was no recurrence of symptoms such as vomiting, abdominal pain, or soft stools.

## Discussion

The dog presented clinical signs of vomiting, chronic soft stools, and abdominal pain, which are commonly associated with a broad range of gastrointestinal disorders, from benign dietary indiscretion to severe life-threatening conditions. The lack of a history of dietary indiscretion or trauma complicated the diagnosis, necessitating a more in-depth evaluation. The persistence of vomiting for 2 weeks raised particular concern, as prolonged vomiting can lead to dehydration and electrolyte disturbances, complicating the clinical assessment (8).

The development of leukocytosis in this case could be due to chronic hematoma, possibly indicating an ongoing inflammatory response, which is typically associated with unresolved or prolonged hematomas. This chronic inflammation may result in a sustained elevation in white blood cell count as the body attempts to manage the injury and repair the tissue over time (9). Platelet levels may either remain normal or show mild thrombocytosis as a response to injury and healing. Blood loss, however, could prevent a significant increase in platelet counts. Dehydration, due to the chronic nature of the condition, may result in an elevation of red blood cell, hematocrit and hemoglobin levels, reflecting fluid loss. While hematoma may cause mild anemia, the chronic progression of the condition could lead to increased hematocrit and hemoglobin levels, a pattern consistent with this case (10).

Thus, based on the hematological evaluation and signs of dehydration, it is likely that the lesion had been present for a prolonged period, as reflected in the history of chronic soft stools and vomiting. These findings may vary depending on the hematoma’s severity and any associated complications, such as secondary infection or fluid shifts (8).

In veterinary practice, when abdominal ultrasonography reveals mixed echogenicity, it can be associated with various conditions. In cases of hematomas, early stages may exhibit a combination of hyperechoic and hypoechoic areas (11). In abscesses, the hypoechoic area generally represents fluid accumulation, while the hyperechoic area may indicate tissue response or inflammation (12). Neoplasms, particularly malignant tumors, may also present with mixed echogenicity due to necrosis or hemorrhage within the tumor (13). Cysts with hemorrhage or infection can show mixed echogenicity due to the presence of blood or infectious material (14, 15). Similarly, fibrosis or scar tissue resulting from previous injuries may demonstrate a mixed echogenic pattern (16). To confirm these diagnoses, further imaging, biopsy, or laboratory testing may be required.

In this case, the owner strongly opposed performing fine needle aspiration cytology due to concerns about the patient’s aggressive behavior and the potential for stress-induced complications such as respiratory distress or hyperthermia. The owner also expressed significant anxiety regarding the risks of sedation or anesthesia in brachycephalic breeds like French Bulldogs. As a result, they declined any additional diagnostic procedures requiring sedation or anesthesia that were not directly related to treatment. However, since histopathological examination of the mass was already planned following surgical removal, the necessity for additional procedures such as fine needle aspiration cytology was considered relatively low.

Based on histopatholgical findings, granulomatous inflammation was observed in the mesentery adjacent to the site of the jejunal intramural hematoma. While granulomatous inflammation is typically associated with infectious, autoimmune, or foreign-body reactions, it may also develop as a secondary response to tissue injury (17). In particular, an intramural hematoma can act as an inert substance that is not readily cleared by the host, thereby inducing a foreign body-type granulomatous reaction. This pattern is consistent with non-infectious granulomatous inflammation and is often accompanied by neovascularization and early fibrosis, supporting the interpretation of a chronic reactive process rather than a primary granulomatous disease (18). Therefore, the mesenteric granulomatous inflammation identified in this case was most likely a secondary response to localized tissue injury caused by the hematoma. This interpretation is further supported by the absence of identifiable infectious organisms on histochemical staining and the lack of clinical or histological evidence of infectious or autoimmune disease.

Histopathological examination confirmed the diagnosis of an intramural hematoma, validating both the diagnostic and therapeutic decisions, particularly in light of the challenging clinical presentation. Given the non-specific nature of the symptoms, radiographic and ultrasonographic imaging were utilized for further evaluation of the gastrointestinal tract. While radiographs revealed food retention in the stomach without significant findings in this case, ultrasonography identified a not well demarcated and heterogeneous mass in the jejunal region, consistent with intramural hematoma (3). This case underscores the limitations of radiographs in detecting certain gastrointestinal issues and highlights the importance of ultrasonography, particularly when initial diagnostic tests are necessary.

According to a prior study, the most common cause of intramural hematoma in the small intestine of children is abdominal trauma (19). The increased risk of jejunal hematomas in children is attributed to anatomical differences, including underdeveloped abdominal musculature and a shallower anteroposterior depth of the abdominal cavity compared to adults (20). Given the patient’s aggressive nature, trauma could not be completely ruled out. However, the owner was highly attentive and careful in managing the patient, and reported no incidents that could have resulted in significant trauma. Moreover, physical examination revealed no external signs of trauma, such as bruising, lacerations, or abrasions. In addition, no trauma-associated lesions were identified in other abdominal organs or the abdominal wall during exploratory laparotomy. These findings collectively rule out trauma as the underlying cause in this case.

The possibility of a transient intussusception that had spontaneously resolved was also considered. Such cases generally present with acute abdominal pain or sudden anorexia due to intermittent obstruction. In contrast, the patient in this case exhibited a more insidious and continuous progression of symptoms, which did not align with the typical episodic clinical pattern associated with self-limiting intussusception (21). Moreover, careful intraoperative inspection of the entire abdominal cavity, including all segments of the gastrointestinal tract, failed to reveal any macroscopic evidence typically observed in prior intussusception, such as segmental bowel wall swelling, mesenteric thickening, or venous and lymphatic engorgement within the affected region (22).

The potential for foreign body-induced intestinal injury was included in the differential assessment. Typically, such trauma is confined to the mucosa and manifests as localized inflammation, mucosal erosion, or ulcer formation (23). However, in this case, histological evaluation did not identify any signs of epithelial disruption or ulceration indicative of mechanical injury. The hematoma was restricted to the muscularis layer, while the mucosal surface showed only vascular congestion and lymphatic dilation without associated tissue damage. These findings are not characteristic of a foreign body-related etiology. The owner was generally attentive to the dog’s behavior and condition and reported no recent episodes of dietary indiscretion or foreign body ingestion.

Other causes of jejunal hematomas include hepatopathy, anticoagulant use, and coagulopathies like hemophilia (24). In this case, based on the owner’s statements and laboratory tests, hepatopathy, trauma, and medication use were ruled out. Moreover, due to normal coagulation testing, coagulopathies could be ruled out as a contributing factor.

While this case had a successful immediate outcome, monitoring over a 1–2 years period would be beneficial to evaluate resolution or recurrence, as suggested by some studies on intramural hematomas (6, 25). However, no recurrence of the hematoma was observed during the 6-month follow-up period after resection in this case.

This case highlights the importance of considering rare conditions like non-traumatic intramural hematomas in the differential diagnosis of prolonged gastrointestinal symptoms, even in animals without a history of trauma or dietary indiscretion. Additionally, it emphasizes the diagnostic value of advanced imaging techniques, particularly ultrasonography, in identifying intestinal wall abnormalities that may be missed on conventional radiography. While CT imaging was not used in this case, it could be considered in future cases for further characterization of the hematoma and potential identification of underlying causes.

In summary, this case describes a rare instance of a non-traumatic intramural hematoma in the jejunum of a 2-year-old neutered male French Bulldog. The dog was presented with non-specific clinical signs, including vomiting, soft stools, and abdominal pain. The owner reported that the dog had consumed only its regular diet and had no history of dietary indiscretion or trauma, nor had any medications that could affect coagulation been administered. These findings underscore the importance of thoroughly investigating gastrointestinal symptoms, even when there are no obvious external risk factors.

## Conclusion

This case underscores the importance of including intramural hematomas in the differential diagnosis for dogs presenting with non-specific gastrointestinal symptoms, even in the absence of trauma and coagulation abnormalities.

## Data Availability

The original contributions presented in the study are included in the article, further inquiries can be directed to the corresponding author.
